# Proteolysis of microtubule associated protein 2 and sensitivity of pancreatic tumours to docetaxel

**DOI:** 10.1054/bjoc.2000.1294

**Published:** 2000-07-24

**Authors:** R Veitia, S David, P Barbier, M Vantard, P Gounon, M C Bissery, A Fellous

**Affiliations:** Unité d’Immunogénétique Humaine, Institut Pasteur, Paris, 75724, France; Department of Biochemistry, University of Havana, Cuba; Laboratoire de Pharmacologie Expérimentale et Clinique, Institut de Génétique Moléculaire, Paris, 75010, France; Laboratoire de Physiologie Cellulaire, CNRS URA 576, CEA, Grenoble, France; Unité de Microscopie Electronique Institut Pasteur, Paris, France; Rhône-Poulenc-Rorer SA, Laboratoire de Cancérologie Expérimentale, Vitry sur Seine, 94403, France

## Abstract

We have studied the state of microtubule associated protein 2 (MAP2) in the pancreatic ductal adenocarcinomas P03 and P02 (sensitive and refractory to docetaxel respectively) since they express the corresponding mRNA and MAP2-related peptides. Immunohistochemical localization showed that in tumour P03 the MAP2-related peptides are highly expressed and confined to the epithelial malignant cells while in P02 the intensity of the immunostaining is lower. However, anti α-tubulin staining followed a similar pattern suggesting that the net amount of macromolecular structures in the sensitive tumour is higher than in the refractory one. This may explain its higher sensitivity to docetaxel, because tubulin assembled into microtubules is the target of the drug. We found that protein extracts from both tumours differed in their proteolytic activity on rat brain MAP2. Since the proteolysis pattern obtained was similar to the one produced by Cathepsin D, we studied the effect of MAP2 proteolysed by this enzyme on microtubule formation in vitro. Proteolysis was found to increase the tendency of tubulin to assemble into macromolecular structures (microtubules and aggregates) in the presence of docetaxel. This suggests that in vivo proteolysis of MAP2 might increase microtubule alterations and potentiate the antitumour effect of docetaxel. © 2000 Cancer Research Campaign


*British Journal of Cancer* (2000) **83**(4), 544–549
© 2000 Cancer Research Campaign
doi: 10.1054/ bjoc.2000.1294, available online at http://www.idealibrary.com on IDEAL®

### Proteolysis of microtubule associated protein 2 and sensitivity of pancreatic tumours to docetaxel

**R Veitia[1,2], S David[3], P Barbier[3], M Vantard[4], P Gounon[5], MC Bissery[6] and A Fellous[3]**

[1]Unité d'Immunogénétique Humaine, Institut Pasteur, 75724 Paris, France; [2]Department of Biochemistry, University of Havana, Cuba; [3]Laboratoire de Pharmacologie Expérimentale et Clinique, Institut de Génétique Moléculaire, 75010 Paris, France; [4]Laboratoire de Physiologie Cellulaire, CNRS URA 576, CEA, Grenoble, France; [5]Unité de Microscopie Electronique Institut Pasteur, Paris, France; [6]Rhône-Poulenc-Rorer SA, Laboratoire de Cancérologie Expérimentale, 94403 Vitry sur Seine, France

**Summary** We have studied the state of microtubule associated protein 2 (MAP2) in the pancreatic ductal adenocarcinomas P03 and P02 (sensitive and refractory to docetaxel respectively) since they express the corresponding mRNA and MAP2-related peptides. Immunohistochemical localization showed that in tumour P03 the MAP2-related peptides are highly expressed and confined to the epithelial malignant cells while in P02 the intensity of the immunostaining is lower. However, anti α-tubulin staining followed a similar pattern suggesting that the net amount of macromolecular structures in the sensitive tumour is higher than in the refractory one. This may explain its higher sensitivity to docetaxel, because tubulin assembled into microtubules is the target of the drug. We found that protein extracts from both tumours differed in their proteolytic activity on rat brain MAP2. Since the proteolysis pattern obtained was similar to the one produced by Cathepsin D, we studied the effect of MAP2 proteolysed by this enzyme on microtubule formation in vitro. Proteolysis was found to increase the tendency of tubulin to assemble into macromolecular structures (microtubules and aggregates) in the presence of docetaxel. This suggests that in vivo proteolysis of MAP2 might increase microtubule alterations and potentiate the antitumour effect of docetaxel. © 2000 Cancer Research Campaign

Docetaxel (Taxotere, TXT) is a semisynthetic taxoid obtained by the chemical modification of a non-cytotoxic terpenoid containing the taxane nucleus extracted from the needles of *Taxus baccata* (Lavelle and Fizames 1989). Preclinical results have revealed a very broad spectrum of antitumour activity against breast, lung, pancreas, ovarian, colon, head and neck carcinomas (Bissery et al, 1995). Clinical results showed high single agent activity against anthracycline refractory breast cancer but also against a variety of other tumours including small cell lung cancer, ovarian, gastric and head and neck carcinomas (Van Oosterom and Schrijvers 1995). Docetaxel, as well as the other taxanes, acts by stabilizing microtubules to levels incompatible with normal metabolism. β-tubulins are the targets for the drugs (Combeau et al, 1994) and previous works have indicated that several β-tubulin isoforms show an overexpression in cell lines resistant to taxol (Haber et al, 1995; Jaffrézou et al, 1995).

We have recently used two pancreatic murine tumours (P03 and P02) as models to study possible determinants of docetaxel sensitivity in vivo. Docetaxel was highly active in vivo against advanced stage pancreatic ductal adenocarcinoma P03 and inactive against early stage P02 (Bissery et al, 1991; Veitia et al, 1998). We showed that the sensitive tumour P03 expresses a higher level of Tau proteins when compared to the docetaxel refractory neoplasm P02 (Veitia et al, 1998). This higher quantity of Tau may increase the sensitivity to the drug since both Tau and docetaxel stabilize microtubules. In this report we concentrate on MAP2 since we have shown that tumours P02 and P03 express the corresponding mRNA and that MAP2-related peptides are recognized by antibodies directed against the brain MAP2 protein (Veitia et al, 1998). In addition, it has been shown that proteolysis of MAP2 alters its interaction with tubulin and promotes tubulin aggregation into anomalous structures in the presence of vinblastine (Fellous et al, 1994). We decided to study the effect of cathepsin D-proteolysed MAP2 on microtubule formation in vitro, in the presence of docetaxel. Cathepsin D was chosen as model protease because its proteolysis pattern is similar to the one produced by tumour (P02 and P03) extracts. In addition, immunological evidence of its presence in these neoplasms was obtained. This type of in vitro approach may provide insights of the contribution of MAP2 proteolysis to the modulation of the malignant cell response to the drug.

*Received 30 November 1999*
*Revised 22 March 2000*
*Accepted 26 March 2000*

*Correspondence to:* R Veitia

### MATERIAL AND METHODS

### Tumour models and mice

Docetaxel was previously evaluated for its antitumour activity in vivo against murine pancreatic ductal adenocarcinomas P03 and P02 and was found highly active against P03 and inactive against P02. The tumours were maintained subcutaneously by serial passages in the mouse strain of origin (C57BI/6) obtained at IFFA CREDO (L'Arbresle, France) from strains obtained from the Jackson Laboratories (Bar Harbor, ME, USA), when needed the tumours were removed and frozen. Tumours are in the National Cancer Institute repository (Frederick, MD, USA).

### Antibodies

Anti α- and β-tubulin are two monoclonals antibodies (N356 and N357) obtained from Amersham (Arlington Heights, IL, USA). Anti-MAP2 152 is a monoclonal produced in our laboratory (Kalil et al, 1988; Veitia et al, 1998). The anti-mouse biotynilated antibody was from DAKO, AS (Denmark). The two anti-tubulin binding domain polyclonal antibodies were prepared against a synthetic peptide chosen in a region of homology between the tubulin binding domains (TBD) of MAP2 and Tau (VTSKIGSLENIKHVPGGG). The peptide was synthesized on a column containing multiple lysine residues. No coupling to a carrier protein was required before immunization. Immunization schedule was as follows: a first subcutaneous injection (2 mg peptide in complete Freund adjuvant) then a second and third intramuscular (IM) injections (1 mg peptide in incomplete Freund adjuvant) on days 10 and 21 respectively. Two additional IM injections were performed on days 36 and 50 (1 mg peptide, incomplete adjuvant). Two antibodies (Prom A and M) reacting differently against the MAP2 fragments containing the TBD were obtained (See Results and Discussion).

### Immunohistochemistry

Paraffin-embedded pancreatic tumours were deparaffined by a xylene-ethanol-water treatment. Endogenous peroxidase activity was inhibited with hydrogen peroxide and nonspecific antibody binding sites were blocked with fetal calf serum. Slices were incubated with anti α-tubulin or anti-MAP2-152 monoclonal antibodies (diluted 1/2000 and 1/500 respectively) overnight at 4°C, and then incubated with an antimouse biotinylated antibody and a complex streptavidin-peroxidase. Detection was carried out using aminoethylcarbazole (AEC, Sigma). Sections were counterstained with haematoxylin, dehydrated and mounted. Images were acquired by a Tri-CDD device (LH750RC3, Lhesa Rungis, France).

### Western blot analysis of pancreatic tumours

Western blots were carried out as described by Veitia et al (1998) using the following antibodies: anti α-tubulin (Sigma), anti MAP2-152, polyclonal anti-Cathepsin D (DAKO) and the two polyclonals anti-TBD PromA and Prom M.

### In vitro MAP2 proteolysis

MAP2 was prepared from 9-day-old rat brains as described by Fellous et al (1977). Proteolysis was induced by either extracts from tumours or cathepsin D. The extracts were prepared by homogenizing 1 g of tumour tissue in 5 ml of 10 mM phosphate buffer pH 5.8. The suspension obtained was sonicated for 30 s and clarified by centrifugation (100 000 ***g*** for 30 min). Proteolysis was carried out at 37°C in the same phosphate buffer using either 1.5 U of cathepsin D or 190 μg of total protein of tumour extract on 80 μg of MAP2. The reaction was stopped at different times (0–60 min) by adding 1/10 volume of 10× microtubule polymerization buffer pH = 6.8 (1 M MES, 10 mM mM EGTA, 1 mM EDTA, 10 mM $MgCl_2$, 10 mM GTP, 10 mM β-mercaptoethanol). This changes the nature of the buffer and corrects the pH to 6.4, necessary for polymerization.

### Microtubule assembly in the presence of docetaxel. Measurement of turbidity, immunoblot analysis and electron microscopy

Rat brain tubulin prepared as described by Weingarten et al (1975) dialysed against polymerization buffer (1×) and proteolysed MAP2 were mixed in ice-cooled spectrophotometer cells (1 mg Tubulin/0.2 mg MAP2). Small aliquots of a docetaxel solution (0.1 mM) were added with energic stirring to the cold mixture to attain 1 or 4 μM final concentration. Cells were then tranferred to a thermostated UVICON spectrophotometer and turbidity change ($OD_{350\ nm}$) at 37°C was measured for 15 min. Then, the reaction mixture was centrifuged at 12000 rpm for 20 min. Pellets and supernatants were kept for further analyses.

Pellets (depolymerized in 100 μl of polymerization buffer on ice) and supernatants of the experiments described above were electrophoresed in 10% PAGE-SDS gels (NOVEX, San Diego, CA) and transferred onto nitrocellulose membranes following the manufacturer's instructions (NOVEX). Immunoblots were performed with the MAP2 monoclonal antibody 152 produced in our laboratory (Kalil et al, 1988), the two polyclonal antibodies recognizing the tubulin binding domain of MAP2 and an anti-β-tubulin. Detection was carried out by Enhanced Chemiluminescence (ECL, Boehringer, Mannheim, Germany).

Small aliquots of the reaction mixtures described above (after polymerization) were also analysed by transmission electron microscopy as describe previously (Fromes et al, 1996).

### RESULTS

### Immunolocalization of polypeptides related to MAP2 in P03 and P02 tumours. Western blot analysis

Immunohistochemistry using the anti-MAP2 monoclonal antibody 152 showed the expression of polypeptides related to this protein in both pancreatic adenocarcinomas (Figure 1). In the docetaxel sensitive tumour (P03) the peptides recognized by the antibody were highly expressed and confined to the epithelial malignant cells (Figure 1C). In contrast, in the refractory tumour P02 the intensity of the immunostaining of the epithelial cells was lower (Figure 1D). However, anti-MAP2 and anti α-tubulin staining (Figure 1A,B) were reproducibly correlated suggesting that the net amount of macromolecular structures (presumably microtubules) in the refractory tumour is lower than in the sensitive one.

As reported previously (Veitia et al, 1998), the amount of the MAP2-related peptides detected by Western blot is different in P03 and P02 tumour extracts. The MAP2 with a similar weight to brain MAP2A and B was not detectable probably because of an intense proteolytic activity of the tumours (Figure 2). Notably, P03 showed a greater amount of a polypeptide of 36–38 kDa, that we had previously thought to contain the TBD (Veitia et al, 1998). Interestingly, Figure 2 shows that a 36–38 kDa fragment was recognized not only by the 152 monoclonal antibody recognizing specifically brain MAP2 but also by one of the anti-TBD antibodies (Prom A).

### In vitro proteolysis of rat brain MAP2 induced by tumour extracts or cathepsin D

Figure 3 shows that HMW rat brain MAP2 can be readily proteolysed by tumour extracts. However, for equal amounts of protein the P03 extract seemed to have higher proteolytic activity. In both cases rat brain MAP2 was fully fragmented within the first 15 min of reaction. In P03, a product of 75 kDa, observed at 15 min, disappeared when reaction proceeded for a further 15 min. However, this band remained virtually unchanged in the reaction

© 2000 Cancer Research Campaign

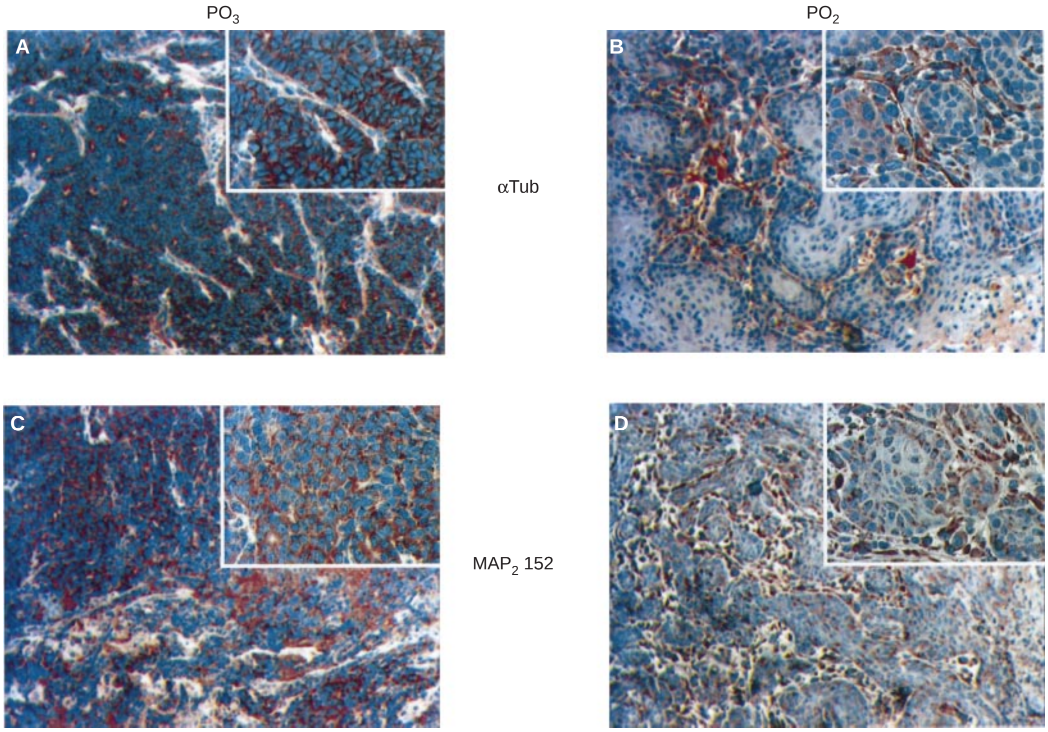

**Figure 1** Immunohistochemical analysis of the pancreatic tumours P03 and P02, sensitive and refractory to docetaxel respectively. Panels **A** and **B**: anti α-tubulin staining (antibody dilution 1/2000). Panels **C** and **D**: anti-MAP2 staining (monoclonal 152, diluted 1/1000). Magnification 150×, insert magnification 600×

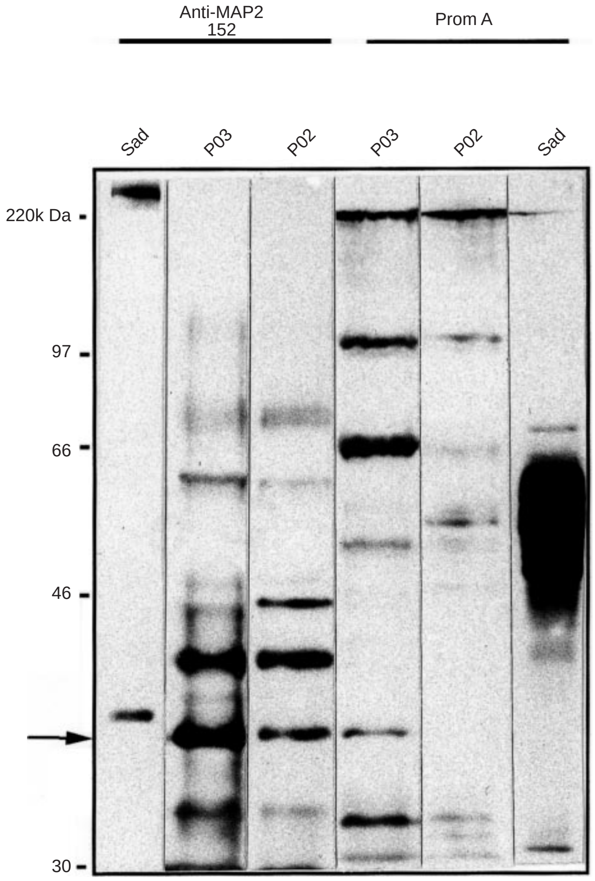

**Figure 2** Western blot of P03 and P02 tumour extracts. Immunodetection was performed on symmetric blots using the anti-MAP2-152 (diluted 1/500) and anti-TBD PromA antibodies (diluted 1/500). Each lane contained equal amounts of α-tubulin. The arrow indicates the 36–38 kDa peptide recognized by PromA antibody

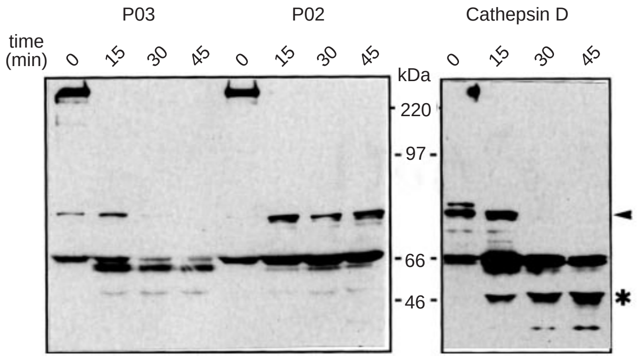

**Figure 3** Proteolysis of rat brain MAP2 induced by P03 and P02 extracts and by Cathepsin D. Eighty μg of MAP2 were treated with 190 μg of tumour extract or 1.5 U of cathepsin D. The arrowhead indicates the 75 kDa fragment mentioned in the text. The asterisk indicates ≈46 kDa fragment(s) produced during proteolysis with P02 extracts even at 45 min of proteolysis. This may point to a difference in the nature or amount of the proteases active against rat brain MAP2 between the tumours.

Western blot analysis of tumour extracts showed the presence of immunoreactive bands with molecular weights corresponding to those expected for cathepsin D itself and its precursor (data not shown). In addition, proteolysis induced by cathepsin D was shown to generate an overall pattern very similar to those obtained after treatment of MAP2 with tumour extracts suggesting that their proteolytic activity may depend to a great extent on cathepsin D or another protease close to it.

 *© 2000 Cancer Research Campaign*

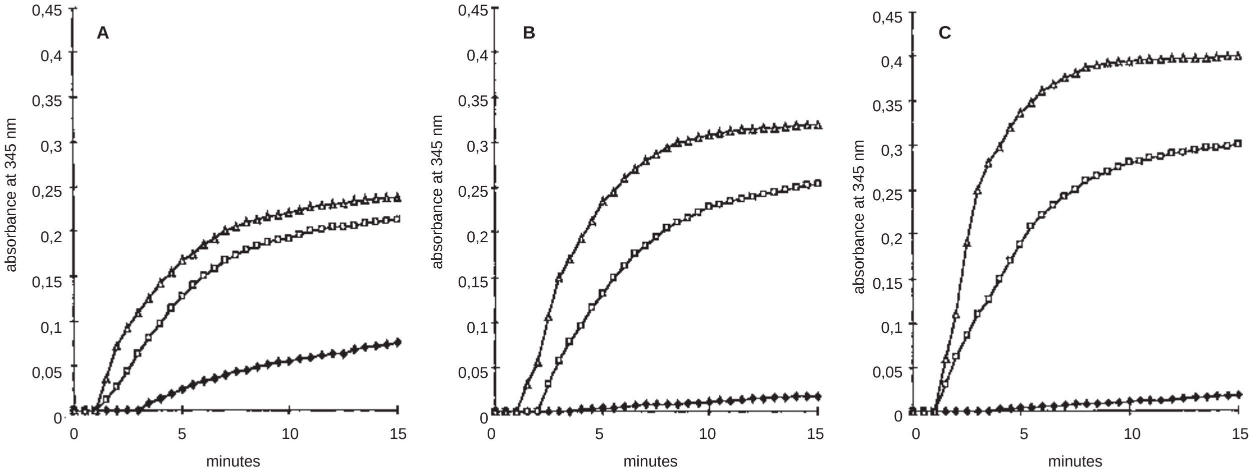

**Figure 4** Effect of MAP2 proteolysis on the kinetics of tubulin polymerization in presence of increasing amounts of docetaxel. One mg/ml of purified tubulin was incubated with MAP2 (0.2 mg/ml) in different degradation states in the absence (solid lozenges) of docetaxel or in the presence of 1 μM (empty squares) or 4 μM (empty triangles) of the drug. Panels **A, B** and **C** present the results for proteolysis times 0, 20 and 40 min respectively

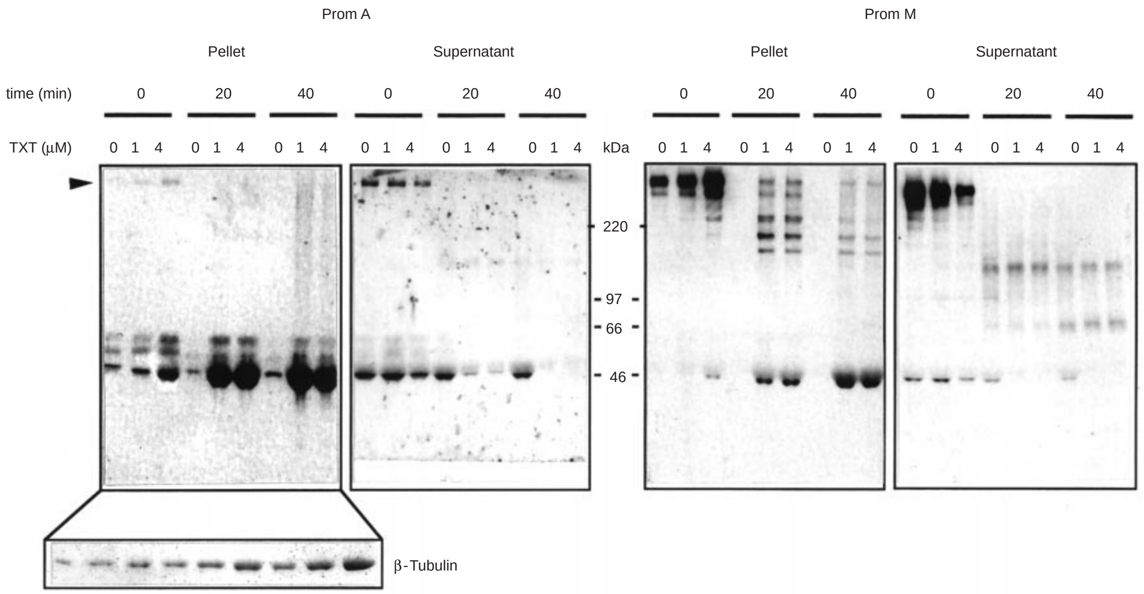

**Figure 5** Western blot analysis of the products obtained after tubulin assembly induced by MAP2 proteolysed for 0 to 40 min and in the presence of increasing amounts of docetaxel (0–4 μM TXT). After incubation of MAP2 (proteolysed or not) and tubulin at 37°C during 15 min the mixtures were centrifuged for 15 min at 12000 ***g***, both the depolymerised pellets and the supernatants were analysed by Western blot (perfectly symmetric) with two different anti-tubulin binding domain antibodies (PromA and B). The 300 kDa protein is indicated by an arrowhead

### Kinetic, Western blot and electron microscopy analyses of tubulin assembly promoted by proteolysed MAP2

The kinetic analysis of tubulin assembly into macromolecular structures using MAP2 with different degrees of proteolysis induced by cathepsin D showed that in absence of docetaxel, the turbidity for any given polymerization time decreased as the protein MAP2 was proteolysed. For docetaxel concentrations 1 and 4 μM there was a clear increase in turbidity when proteolysis time increased from 0 to 40 min (Figure 4). Microtubules from each reaction condition were further studied by both Western blot and electron microscopy.

Western blot analysis of the microtubule pellets with two different antibodies directed against the MAP2/Tau TBD revealed, before effective proteolysis took place, the 300 kDa band as well as minor degradation products (Figure 5). Notice that PromA recognizes poorly the 300 kDa protein in the pellets (faint bands in presence of docetaxel). The intensity of all bands increased as a function of the concentration of docetaxel in a dose-dependent manner. At 20 min of proteolysis, the 300 kDa band almost disappeared and there was a large increase in the amount of degradation fragments (mainly seen when docetaxel was present in the reaction medium). A major degradation product of about 46 kDa accumulated in the polymer phase (microtubule pellet). From a

© 2000 Cancer Research Campaign

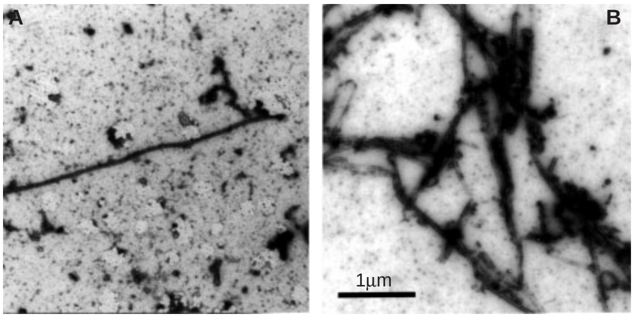

**Figure 6** Electron microscopic analysis of the tubulin polymerization products induced by MAP2 proteolysed for 20 min either in the absence (left panel) or in the presence (right panel) of docetaxel (4 μM)

quantitative point of view, the Western blot results for the supernatants were the reverse of those obtained for the pellets (Figure 5). Notice here that PromA antibody recognized better the 300 kDa MAP2 in the supernatant. The band recognized may represent a different MAP2 isoform (conformation or post-translational modification). The amount of β-tubulins in the pellets (monoclonal antibody N356) increased following the degree of MAP2 proteolysis even in absence of the drug, contrasting with the results of the kinetic studies. This is probably due to the formation of small aggregates that do not alter turbidity but precipitate during centrifugation. The increase in the pellet of both tubulin and some MAP2 fragments, mainly the 46 kDa polypeptide, in the presence of docetaxel showed clearly that MAP2 proteolysis enhanced the assembly of tubulin into macromolecular structures. This observation was confirmed by electron microscopy analysis of the microtubules obtained for each of the reaction conditions. When MAP2 was proteolysed, microtubules were hardly polymerized in the absence of the drug (Figure 6A), whereas at 4 μM docetaxel, a considerable amount of microtubules (normal or open) as well as a net increase in the number of aggregates (dark structures) was noticed (Figure 6B).

### DISCUSSION

Immunohistochemistry of tumours P03 and P02, sensitive and refractory to docetaxel respectively, has shown that both MAP2 related polypeptides and α-tubulin are more abundant in the docetaxel sensitive tumour. This reflects that the net amount of macromolecular structures (presumably microtubules) in the sensitive tumours is higher than in the refractory one. Since docetaxel binds preferentially to tubulin subunits assembled into microtubules rather than to tubulin dimers (Parness and Horwits, 1981), a higher density of the microtubule network in tumour P03 may explain its higher sensitivity to the drug. In addition, microtubules in P03 could be intrinsically more stable than in P02 because for equal amounts of tubulin protein, greater amounts of the microtubule-stabilizing Tau proteins are found in the P03 (Veitia et al, 1998). In addition to this, we decided to explore other factors than microtubule density that may modulate microtubule sensitivity to docetaxel.

Immunoblot analysis of tumour extracts using a monoclonal anti MAP2 antibody has shown the presence of MAP2-related peptides in both tumours P03 and P02. An increase in the amounts of a 36–38 kDa product thought to contain the TBD has also been noticed in the docetaxel sensitive P03 (Veitia et al, 1998). Interestingly, the polyclonal anti-TBD (PromA) is able to recognize a band of the same length in P03 extracts. This fragment is likely to be a MAP2 degradation product. However, we cannot exclude the possibility that it is a comigrating Tau fragment also containing the TBD. To address this question immunoprecipitation was unsuccessfully attempted. In any case, one may suppose that MAP2 and Tau TBDs share not only structural but also functional similarities.

The study of the proteolytic activity of tumour extracts (P03 and P02) on purified rat brain MAP2, showed that both extracts were able to degrade the MAP although P03 displayed a greater activity (for equal amounts of total protein) (Figure 3). The kinetic analysis of the influence of proteolysis induced by the extracts on the capability of MAP2 to promote tubulin assembly into macromolecular structures was attempted. However, the presence of some component in the extract produced anomalous results (lack of plateau for the kinetic curves). Figure 3 shows that the degradation patterns produced by the tumour extracts and by cathepsin D are very close. In addition, anti-cathepsin D immunoreactive polypeptides were found in both tumours (P02 and P03). Therefore, cathepsin D was chosen as a model protease to further study the effect of docetaxel on microtubule assembly promoted by proteolysed MAP2.

The kinetic monitoring of tubulin polymerization shows that in the absence of docetaxel, the ability of MAP2 to induce microtubule formation is impaired as it is proteolysed by cathepsin D (Figure 4). Addition of the drug causes a notable increase in turbidity even when the MAP is proteolysed, suggesting that tubulin polymers are produced. Interestingly, this increase in OD is more important as MAP2 is degraded suggesting that, in addition to microtubule polymerization, a concomitant aggregation process induced by the drug is also taking place. Electron microscopy has shown that it is indeed the case (Figure 6). It is worth noting that although information concerning intracellular concentrations of docetaxel is scarce (Lavelle et al, 1995), rough calculations suggest that 1–4 μM (as used in our study) can be relevant at least in cultured cells.

Western blots from the kinetic experiments show that either the whole MAP or its proteolytic products including the 46 kDa fragment can promote tubulin polymerization and/or aggregation and suggest that docetaxel enhances the recruitment of MAP2 fragments which have an impaired ability to induce tubulin assembly into macromolecular structures. Interestingly, the anti-TBD antibody PromM recognizes very well the HMW fragments of MAP2 while PromA is less effective in doing so. This suggests that, MAP2 being a very huge protein, the PromA epitope can be masked by another portion of the protein even under mild denaturing conditions. These data suggest that MAP2 proteolysis induces conformational changes in the protein around the TBD. A possible interaction between the MAP2 projection arm and the TBD that could affect the conformation or accessibility of the latter. The conformational changes involving the TBD might favour binding to tubulin in a metastable way which would then be stabilized by the drug. However, some of the TBD-containing fragments may be impaired to promote efficient microtubule polymerization and would still induce tubulin aggregation. The latter process should be enhanced by the presence of docetaxel. Alternatively, changes in tubulin upon binding of MAP2 may depend on the nature of the proteolytic fragment. In this case, the complexes tubulin-MAP2 may have different affinities for docetaxel or may more or less easily produce abnormal structures.

 *© 2000 Cancer Research Campaign*

Proteolysis of MAP2 may take place in a poorly controlled way in the neoplastic cells and may induce changes in MAP2 conformation. These changes may increase sensitivity of the tumour to docetaxel either by increasing the density of the microtubule network and/or the amount of abnormal tubulin polymers. This mechanism was suggested to explain the higher sensitivity of MAP2 microtubules to vinblastine and other microtubule agents (Fellous et al, 1994). The identification of other neoplastic proteases that are able to degrade MAP2 in tumours P02 and P03 has to be explored. We suggest that cathepsin D-like proteases and cathepsin D itself may degrade MAP2 in vivo and contribute to modulate tumour response to docetaxel.

### ACKNOWLEDGEMENT

RV has been supported by the Société de Secours des Amis des Sciences.

### REFERENCES

Bissery MC, Guenard D, Gueritte-Voegelein F, Lavelle F (1991) Experimental antitumor activity of Taxotere (RP56976, NSC 628503), a taxol analogue. *Cancer Res* **51**: 4845–4852

Bissery MC, Nohynek G, Sanderink G-J, Lavelle F (1995) Docetaxel (Taxotere): a review of preclinical and clinical experience. Part I: preclinical experience. *Anti-cancer Drugs* **6**: 339–355

Combeau C, Commerçon A, Mioskowski C, Rousseau B, Aubert F, Goldner M (1994) Predominant labeling of β over α tubulin from porcine brain by a photoactivable taxoid derivative. *Biochemistry* **33**: 6676–6683

Fellous A, Francon J, Lennon AM, Nunez J (1997) Microtubule assembly in vitro. Purification of assembly promoter factors. *Eur J Biochem* **78**: 167–174

Fellous A, Prasad V, Ohayon R, Jordan MA, Luduena RF (1994) Removal of the projection domain of microtubule-associated protein 2 alters its interaction with tubulin. *J Prot Chem* **13**: 381–391

Fromes Y, Gounon P, Veitia R, Bissery MC, Fellous A (1996) Influence of Microtubule-associated proteins on the differential effects of paclitaxel and docetaxel. *J Prot Chem* **15**(4): 377–388

Haber M, Burkhart CA, Lee Regl D, Madafiglio J, Norris MD, Horvitz SB (1995) Altered expression of Mβ2, the class ii β-tubulin isotype, in a murine J774.2 cell line with a high level of taxol resistance. *J Biol Chem* **270**(52): 31269–31275

Jaffrézou J-P, Dumontet C, Derry WB, Duran G, Chen G, Tsuchiya E, Wilson L, Jordan MA, Sikic BI (1995) Novel mechanism of resistance to Paclitaxel (taxol) in human K562 leukemia cells by combined selection with PSC 833. *Oncol Res* **7**(10/11): 517–527

Kalil J, Fellous A, Fellous M (1988) Applications de I’immunilogie aux cultures in vitro. In *Culture de cellules animales: Methodologies et applications*. Adolphe M and Barlovats-Meimon G (eds). pp. 101–130. Les Editions INSERM, Paris.

Lavelle F, Fizames C (1989) Experimental properties of RP56976, a taxol derivative. *Proc. AACR* **30**: 566

Lavelle F, Bissery MC, Combeau C, Riou JF, Vrignaud P, Andre S (1995) Preclinical evaluation of Docetaxel (Taxotere). *Seminars in Oncology* **22**(2), Suppl. 4, 3–16

Parness J, Horwits SB (1981) Taxol binds to polymerized tubulin in vitro. *J Cell Biol* **91**: 479–487

Van Oosterom AT, Schrijvers D (1995) Docetaxel (Taxotere): a review of preclinical and clinical experience. Part I: preclinical experience. *Anti-cancer Drugs* **6**: 356–368

Veitia R, Bissery MC, Martinez C, Fellous A (1998) Tau expression in murine adenocarcinomas correlates with docetaxel sensitivity in tumor-bearing mouse. *British J Cancer* **78**(7): 871–877

Weingarten MD, Lockwood AH, Hwo SY, Kirschner MW (1975) A protein factor essential for microtubule assembly. *Proc Natl Acad Sci USA* **72**: 1848–1852

© 2000 Cancer Research Campaign
